# Novel Cure for Phthisis, and a New Operation of Castration

**Published:** 1858-09

**Authors:** 


NOVEL CURE FOR PHTHISIS, AND A NEW OPERATION OF CASTRATION.—In one of our Exchanges, *La Iberia Medica*, January 10th, 1858, will be found, under the caption of "Cure of Phthisis by Lightning," the details of a remarkable case, reported by Don José Otero y Ortiz, of San Cristóbal, Cuba. The following is an abstract of this case, which may be supposed to be merely an emanation from a lively imagination; or, it may be, that Spain is endeavoring to rival America in remarkable cases. "Credat Judæus Appella, non ego."

A man, thirty-one years of age, suffering from pulmonary consumption, characterized by hæmoptysis, expectoration of purulent matter, aphonia, and hectic fever, was sitting in a house of palm-leaves playing on a guitar, when he heard a loud noise and felt a hard blow on the back part of his left arm, causing him to fall to the ground, losing his power of motion, but not his consciousness. At the same moment he observed a small bluish-white globe of fire advancing toward him, which entered the sleeve of his right arm, ascended to the shoulder, crossed over the neck to the left shoulder, and then ran down his left side to his hips, pubis, and genital organs, and left his thigh for the house, and melted the metals and destroyed the furniture. The patient observed thirteen large burns upon his body; his testicles had disappeared, and his penis had become reduced to a very small size. The burns had cicatrized in three months, and in proportion as the weakened state of the system was ameliorated did the pulmonary symytoms disappear. At the end of six months he had entirely recovered; his voice had become very soft and fine; his beard was falling out, and he presented all the external appearances of a eunuch. The cure is ascribed to "the revulsive effect produced by the extensive burns, or, perhaps, to some particular virtue possessed by electricity." Should our Cuban brethren believe such to be the case, we would advise them to attach their consumptive patients to a lightning rod during a thunder-storm, so that they may the better obtain the "particular virtue" of electricity.

